# Virtual Monoenergetic Images of Dual-Energy CT—Impact on Repeatability, Reproducibility, and Classification in Radiomics

**DOI:** 10.3390/cancers13184710

**Published:** 2021-09-20

**Authors:** André Euler, Fabian Christopher Laqua, Davide Cester, Niklas Lohaus, Thomas Sartoretti, Daniel Pinto dos Santos, Hatem Alkadhi, Bettina Baessler

**Affiliations:** 1 Institute of Diagnostic and Interventional Radiology, University Hospital Zurich, University of Zurich, Raemistrasse 100, 8091 Zurich, Switzerland; andre.euler@usz.ch (A.E.); fabian.laqua@online.de (F.C.L.); davide.cester@usz.ch (D.C.); niklas.lohaus@usz.ch (N.L.); thomas.sartoretti@usz.ch (T.S.); hatem.alkadhi@usz.ch (H.A.); 2 Medical Faculty, University Hospital Cologne, Institute of Diagnostic and Interventional Radiology, University of Cologne, Kerpener Str. 62, 50937 Cologne, Germany; daniel.pinto-dos-santos@uk-koeln.de

**Keywords:** radiomics, spectral CT, dual-energy CT, oncology, machine learning, computed tomography, reproducibility, test–retest, virtual monoenergetic reconstructions

## Abstract

**Simple Summary:**

Virtual monoenergetic images from dual-energy CT are incrementally used in routine clinical practice. Thus, radiomic analysis will be more often performed on these images in the future. This study characterized the test–retest repeatability and reproducibility of radiomic features from virtual monoenergetic images and their impact on machine-learning-based lesion classification. The results of this study provide a basis to improve radiomic analyses and identify the role of feature stability in classification tasks when using virtual monoenergetic imaging with different scan or reconstruction parameters in multicenter clinical studies.

**Abstract:**

The purpose of this study was to (i) evaluate the test–retest repeatability and reproducibility of radiomic features in virtual monoenergetic images (VMI) from dual-energy CT (DECT) depending on VMI energy (40, 50, 75, 120, 190 keV), radiation dose (5 and 15 mGy), and DECT approach (dual-source and split-filter DECT) in a phantom (ex vivo), and (ii) to assess the impact of VMI energy and feature repeatability on machine-learning-based classification in vivo in 72 patients with 72 hypodense liver lesions. Feature repeatability and reproducibility were determined by concordance–correlation–coefficient (CCC) and dynamic range (DR) ≥0.9. Test–retest repeatability was high within the same VMI energies and scan conditions (percentage of repeatable features ranging from 74% for SFDE mode at 40 keV and 15 mGy to 86% for DSDE at 190 keV and 15 mGy), while reproducibility varied substantially across different VMI energies and DECTs (percentage of reproducible features ranging from 32.8% for SFDE at 5 mGy comparing 40 with 190 keV to 99.2% for DSDE at 15 mGy comparing 40 with 50 keV). No major differences were observed between the two radiation doses (<10%) in all pair-wise comparisons. In vivo, machine learning classification using penalized regression and random forests resulted in the best discrimination of hemangiomas and metastases at low-energy VMI (40 keV), and for cysts at high-energy VMI (120 keV). Feature selection based on feature repeatability did not improve classification performance. Our results demonstrate the high repeatability of radiomics features when keeping scan and reconstruction conditions constant. Reproducibility diminished when using different VMI energies or DECT approaches. The choice of optimal VMI energy improved lesion classification in vivo and should hence be adapted to the specific task.

## 1. Introduction

Radiomics has been investigated for its use as a biomarker in disease characterization and for the assessment of oncologic treatment response [1,2,3,4], predicting treatment efficacy [5], and patient outcome [6,7,8]. In radiomics, morphological, intensity-based, and textural features are assessed to reveal imaging patterns that go beyond the traditional, sole visual image interpretation.

Dual-energy computed tomography (DECT) has increasingly been used in oncologic imaging to improve the detection and conspicuity of malignant lesions [9,10,11]. DECT enables the reconstruction of virtual monoenergetic images (VMI), which have been shown to improve lesion detection at low keV levels [10]. This is achieved by increasing the lesion-to-background contrast-to-noise ratio based on an increase in the CT attenuation of iodinated structures. A recent white paper of the Society of Computed Tomography and Magnetic Resonance [12] and a multi-institutional consensus [13] have advocated for the routine use of VMI at 50 keV (i.e., for high contrast) and 70 keV (i.e., for low noise) in DECT of the abdomen [13].

First studies have shown the potential benefit of radiomics alone or in combination with machine learning when applied to images obtained with DECT [14,15,16,17,18]. However, the increased use of VMI in routine oncological imaging along with radiomics analysis requires standardization, repeatability, and reproducibility of radiomic features [19] to ensure patient safety and to avoid misinterpretation of results, especially if they are used in the course of tumor treatment response assessment. Yet, it is unknown whether the classification performance is influenced by these factors.

Previous studies have reported that radiomic features are highly affected by CT acquisition and reconstruction settings [20,21,22,23,24,25]. To date, only a few studies have investigated the impact of VMI on radiomics in the liver [26], for cervical lymphadenopathy [16,17], and benign parotid tumors [15]. However, these investigations were limited to a single DECT approach (fast kVp-switching DECT), a single organ, and to the assessment of first-order features only [16,26]. Thus, further research is required to define the impact of different energy levels of VMI as well as different scan settings and other DECT approaches on the repeatability and reproducibility of radiomic features, including higher-order textural features. In addition, it is unclear how radiologic features derived from VMI at different energy levels might affect classification tasks by machine learning algorithms.

Thus, the purpose of our combined phantom and in vivo study was to evaluate the repeatability and reproducibility of radiomic features in VMI at different energies and to assess their impact on machine-learning-based classification as a function of VMI energy, radiation dose, and DECT approach with different CT scanners.

## 2. Materials and Methods

### 2.1. Part I—Ex Vivo Study

The aim of the ex vivo phantom study was to systematically evaluate the repeatability and reproducibility of radiomic features and to assess the impact of feature repeatability and reproducibility on machine-learning-based classification as a function of VMI energy, radiation dose, and DECT approach.

#### 2.1.1. Phantom and Scan Setup

**Figure 1:**
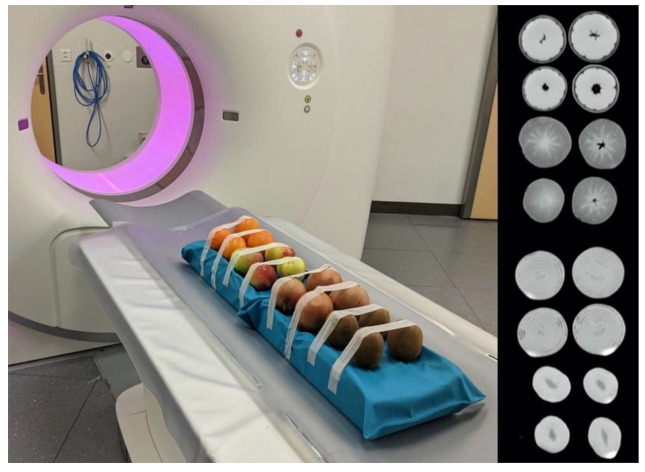
Phantom setup. Photograph (**left**) and axial CT image (**right**) of the phantom consisting of four kiwifruits, four onions, four apples, and four oranges simulating different CT attenuation and tissue textures.

The phantom consisted of a total of four kiwifruits, four onions, four apples, and four oranges. These objects were chosen to reflect different shapes and tissue textures. Their CT attenuation was in the range of tissues typically found in diagnostic imaging in humans at 120 kV, with values resembling fat (apple, mean -179 HU; onion, mean -29 HU), water (orange, mean -5 HU), and soft tissue (kiwifruit, mean 45 HU) [27]. Objects were placed on a radiolucent cushion used in daily routine scanning (Figure 1).

The phantom was imaged in dual-energy mode on (i) a dual-source dual-energy CT (DSDE; SOMATOM Force, Siemens Healthineers, Forchheim, Germany) at a tube voltage of 100 kV and Sn150 kV, and (ii) a single-source split-filter dual-energy CT (SFDE) at AuSn120 kV (SOMATOM Edge Plus, Siemens). Scans were performed at two radiation dose levels with a volume CT dose index (CTDI_vol_) of 5 and 15 mGy. To assess test–retest repeatability, scans were repeated after repositioning the phantom. Vendor-predefined protocols for the abdomen were applied to both DECT systems: collimation of 32 × 0.6 mm², gantry rotation time of 0.5 s, and pitch of 0.3. For each scan setup, VMI at energy levels of 40, 50, 75, 120, and 190 keV were reconstructed (Monoenergetic +, Syngo.via VB50A, Siemens Healthineers) with a slice thickness of 1 mm, a Qr40 kernel, and an advanced modeled iterative reconstruction algorithm at a strength level of 3 (ADMIRE).

#### 2.1.2. Image Segmentation and Feature Extraction

The images of all VMI energy levels were loaded simultaneously into an open-source segmentation software (3D Slicer; version 4.10.1). A semi-automatic grow-from-seeds algorithm was applied to segment a 3D volume of interest (VOI) for each fruit on 120 keV images. No manual correction was performed. The resulting 3D segmentation was then copied onto the geometrically identical images of the remaining VMI energies.

The 3D segmentations underwent a standardized pre-processing to enable standardized feature extraction [19]: spatial resampling to 1 × 1 × 1 mm³ using a Lagrangian polynomial of degree 5; intensity discretization to a bin width of 25; and relative intensity rescaling by using a scale of 500. After standardization, a total of 1218 radiomic features were extracted for each dataset using the pyRadiomics package [28]. Most features in the pyRadiomics package are in compliance with feature definitions as described by the Imaging Biomarker Standardization Initiative (IBSI) [19]. 

Radiomic features corresponded to 7 different matrices/feature classes: first order statistics/histogram matrix, shape-based features, gray level co-occurrence matrix (GLCM), gray level run-length matrix (GLRLM), gray level size-zone matrix (GLSZM), neighboring gray tone difference matrix (NGTDM), and gray level dependence matrix (GLDM) (Supplementary Table S1).

#### 2.1.3. Analysis of Feature Repeatability and Reproducibility

Repeatability was assessed using the calculation of concordance correlation coefficients (CCCs) and dynamic range (DR) across all objects [29,30]. Here, the normalized DR for a feature was defined as $1-\frac{range}{average\;difference}$ [29,31]. Values close to one indicate that the feature has a large biological range with good reproducibility. The percentage of features with a CCC of ≥0.90 was calculated for varying VMI energies, DECT approaches, and radiation doses. 

A cutoff of CCC ≥0.90 was applied to the measures of intra- and inter-scanner feature reproducibility for all VMI energies and radiation doses to indicate feature reproducibility [32]. Percentages of repeatable features were compared using repeatable one-way analyses of variance (ANOVA) with Tukey-type comparisons to correct for multiple testing. 

Statistical analysis was performed in R (version 3.5.3; R Foundation for Statistical Computing, Vienna, Austria) with RStudio (version 1.2.5033; RStudio, Boston, MA, USA [33] using the packages tidyverse [34], summarytools [35], ggplot2 [36], DescTools [37], and multcomp [38].

#### 2.1.4. Impact on Machine Learning Classification

Two different classification approaches were used. Multivariable logistic regression models with combined L1 and L2 penalty (‘elastic net’) [39] and random forests (for sensitivity analysis) were trained to classify the type of fruit. We compared the models (i) by VMI energy (40, 50, 70, 90, 120, 190 keV), (ii) by dual-energy approach (DSDE, SFDE), (iii) by radiation dose (5 or 15 mGy), and (iv) by the three subsets of included features (‘all features’; ‘repeatable features’, CCC ≥ 0.9; and ‘non-repeatable features’, CCC < 0.9). Model evaluation was performed in a tournament-leave-pair-out cross-validation approach [40]. Based on an identification number that uniquely identifies each fruit, these objects were divided in every possible unique combination of a training set and a pair of testing samples. Then, all models were trained and evaluated on each training set and the corresponding test pair; each repetition is called a “tournament” and a tournament is “won” when a certain subject from the test set has a higher predicted score to belong to a certain category than the other one. This procedure was repeated for every unique set of test pairs. The ranking of the number of won tournaments per object was used as a score for the calculation of unbiased concordance statistics (receiver operating characteristic—ROC, and area under the ROC curve—AUC). AUCs were hence calculated in a one-versus-rest manner for every class and then averaged across the classes. The Brier score (a quadratic scoring rule—BS), which also takes into account calibration of the models (i.e., how well the predicted probability for a class matches the actual observed frequency), was used as an additional evaluation metric for the classification tasks. A scaled variant of BS was calculated by $R^{2}\;=\;1-\frac{BS}{BS_{max}}$ and gave the fraction of reduced prediction error compared to a (in this case non-informative) benchmark model.

This evaluation procedure was combined with a bootstrap correction of the bias induced by testing multiple hyperparameter configurations [41] in a random search approach, optimizing the Brier score. This Machine Learning workflow was implemented using an open-source programming language (Python 3.7, using the scikit-learn 0.21.3 package [39]).

### 2.2. Part II—In Vivo Study

The aim of our patient study was to estimate the impact of VMI energy and the selection of repeatable and non-repeatable radiomic features on machine learning classification for a relatively simple clinical classification task (i.e., differentiation between three etiologies of hypodense liver lesions, namely cysts, hemangiomas, and metastases). The primary aim of our study was not to build and validate a classification model with a translational perspective, but rather to use this classification task as an example use case to demonstrate the impact of feature repeatability and choice of VMI on an exemplary machine learning classification model.

#### 2.2.1. Patient Population and Image Reconstruction

This retrospective study was compliant with the Health Insurance Portability and Accountability Act (HIPPA) and approved by the ethics committee (*blinded for review*) under the approval number *blinded for review*. The requirement for study-related informed consent was waived.

**Figure 2:**
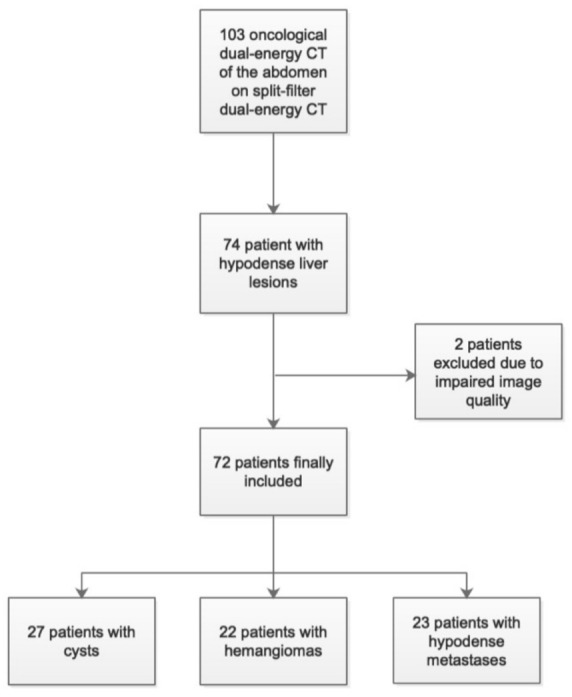
Patient study inclusion flowchart.

We retrospectively identified consecutive oncological patients who underwent a clinically indicated contrast-enhanced DECT of the abdomen in the portal-venous phase between August 2018 and August 2019 on the same single-source split-filter dual-energy CT as used in the phantom study (*n* = 103) (see study flow chart in Figure 2). All scans were reviewed by a board-certified radiologist with 9 years of experience in abdominal imaging and the radiological reports were additionally consulted to ascertain and confirm diagnoses or radiological findings. Only patients with hypodense liver lesions were included (*n* = 74). Exclusion criteria were inadequate image quality due to breathing or motion artefacts (*n* = 2). Finally, 72 patients were included.

The reader selected one hypodense liver lesion per patient and classified the lesion as being either a cyst, hemangioma (atypical hemangiomas were not considered), or metastasis based on CT attenuation, contrast media characteristics, and results of prior and follow-up imaging, also taking into account lesion size dynamics and results from other imaging modalities (including MRI and PET-CT). Within 6 months of the CT examination of interest, 20 patients had received CT imaging only, 13 patients had received CT imaging and MRI, 4 patients had received CT imaging, MRI, and PET-CT imaging, 1 patient had received PET-CT imaging only, and 1 patient had received MRI only. A total of 5 patients had received biopsies for suspicion of metastasis. 

Lesions were classified as 27 cysts (group A), 22 hemangiomas (group B), and 23 metastases (group C). Lesion size was 10.8 ± 19.1 cm^3^ for cysts, 4.4 ± 7.3 cm^3^ for hemangiomas, and 22.2 ± 34.3 cm^3^ for metastases (*p* = 0.01 for cysts vs. hemangiomas and *p* < 0.001 for cysts vs. metastases and for hemangiomas vs. metastases). Patient age was 71 ± 11 years, 61 ± 12 years, 65 ± 12 years for group A, B, and C, respectively. Further information on the study cohort can be found in Table 1.

Scan parameters were identical to those described in the phantom study. For each included case, VMI at energy levels of 40, 50, 75, 120, and 190 keV were reconstructed with the same slice thickness, kernel, and iterative reconstruction algorithm (ADMIRE, level 3) as mentioned in part I.

#### 2.2.2. Image Segmentation and Feature Extraction

Lesion segmentation was manually and independently performed by two readers (one radiology resident with two years of experience and one research assistant with three years of experience in medical imaging) using the same open-source software tool as used in the phantom study. In detail, for each patient, the images of all VMI energies were loaded simultaneously into the software. Then, by consensus, the readers determined the VMI energy that exhibited the optimal lesion visualization. Afterwards, both readers individually and manually performed a 3D segmentation of the lesion. This 3D segmentation was copied onto the anatomically and geometrically identical images of the remaining VMI energies. All 3D segmentations underwent the same standardized pre-processing procedure and feature extraction procedure as described in the phantom study.

#### 2.2.3. Impact on Machine Learning Classification

The modeling procedure was identical to the one used for the phantom study. However, the model comparison was limited to the different VMI energies because rescanning the patients at different radiation doses or on different CT scanners would have been unethical due to the increased radiation exposure. The impact of the same three groups of radiomic features on classification (‘all features’; ‘repeatable features’, CCC ≥ 0.9; and ‘non-repeatable features’, CCC < 0.9) were investigated.

## 3. Results

### 3.1. Part I—Ex Vivo Study

#### 3.1.1. Test–Retest Repeatability of Radiomic Features

**Figure 3:**
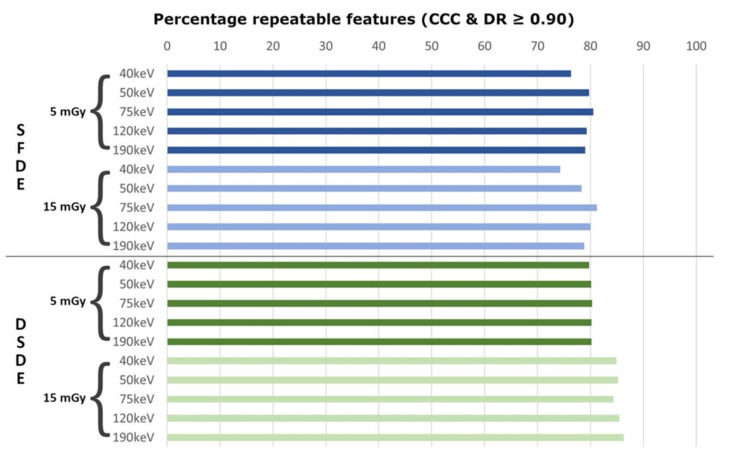
Test–retest repeatability of radiomic features. Bar plots demonstrate the percentage of repeatable features (concordance correlation coefficient (CCC) ≥ 0.9 and dynamic range (DR) ≥ 0.9) for individual VMI energies (40 to 190 keV), DECT approaches (SFDE = split-filter dual-energy, DSDE = dual-source dual-energy), and radiation doses (5, 15 mGy). Overall, DSDE demonstrated higher repeatability at higher radiation dose (light green) and less variability among VMI energies compared to SFDE (blue).

Overall, the percentage of stable features with large biological range (CCC and DR ≥ 0.9) in test–retest assessment differed only slightly among the different VMI energies and scan settings (Figure 3). Significant differences in the number of stable features were observed for DSDE at the 15 mGy radiation dose only (mean percentage of stable features across all VMI energies for DSDE with 15 mGy, 85.2 ± 0.7% vs. DSDE with 5 mGy, 80.1 ± 0.2% (*p* < 0.001) vs. SFDE with 15 mGy, 78.5 ± 2.6% (*p* < 0.001) vs. SFDE with 5 mGy, 79.0 ± 1.6% (*p* < 0.001)). There were no significant differences between mean percentages for DSDE at 5 mGy and SFDE at 5 and 15 mGy.

For SFDE at both radiation doses, variation in the percentage of stable features between the different VMI energies was higher than for DSDE, with a lower percentage of stable features at 40 keV (76% at 5 mGy and 74% at 15 mGy; Figure 3). No relevant impact of VMI energy was found for DSDE at both radiation doses. Overall, the highest percentage of stable features were found for DSDE at 15 mGy and the 190 keV level (*n* = 1051/1218, 86%).

#### 3.1.2. Reproducibility of Radiomic Features

**Figure 4:**
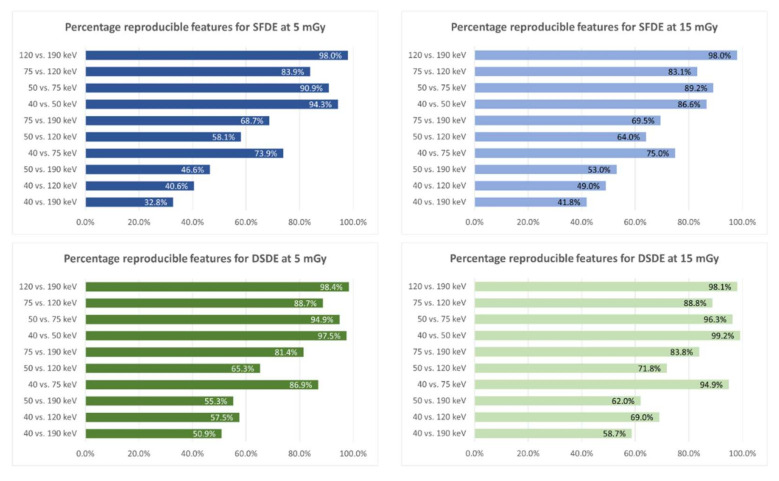
Reproducibility of radiomic features between different VMI energies. Bar plots showing the percentage of reproducible features (CCC ≥ 0.90) for all comparisons among VMI levels as a function of DECT scanner and radiation dose. The percentage of reproducible features decreased with increasing difference in keV level among VMI for both scanners. No major differences could be observed between the two radiation doses (<10%).

In addition to test–retest repeatability, feature reproducibility was assessed between (i) the different VMI energies, (ii) the two radiation doses, and (iii) the two DECT approaches. The percentage of reproducible features between different VMI energies differed substantially (Figure 4). No major differences were observed between the two radiation doses (<10%) in all pair-wise comparisons. At equal VMI energies, reproducibility of features between both DECT approaches ranged between 73–84% and 67–83% at 5 and 15 mGy, respectively (Supplementary Figure S1). The highest percentage of reproducible features was achieved at VMI energies of 75 keV at both radiation doses (83% and 84%, respectively), whereas 40 keV delivered the lowest percentage of reproducible features (73% and 67%, respectively). For both DECT approaches, the percentage of reproducible features was high (>80%) between the two radiation doses (Supplementary Figure S2). The lowest values were achieved at 40 keV (82% for SFDE and 93% for DSDE); the highest at 75 keV (96% for SFDE, and 97% for DSDE).

#### 3.1.3. Impact on Machine Learning Classification

Classification performance was perfect in terms of discrimination (AUC > 0.999) and absolute prediction error (R^2^ = 1.0) if all features or if only repeatable features were used (Supplementary Table S2). For the non-repeatable feature subset, the performance was lower, both in terms of discrimination and prediction error, and differed by the VMI energy (worst performance for 75 keV using SFDE at 15 mGy; R^2^ = 0.52, AUC = 0.96).

### 3.2. Part II—In Vivo

Impact on Machine Learning Classification

**Figure 5:**
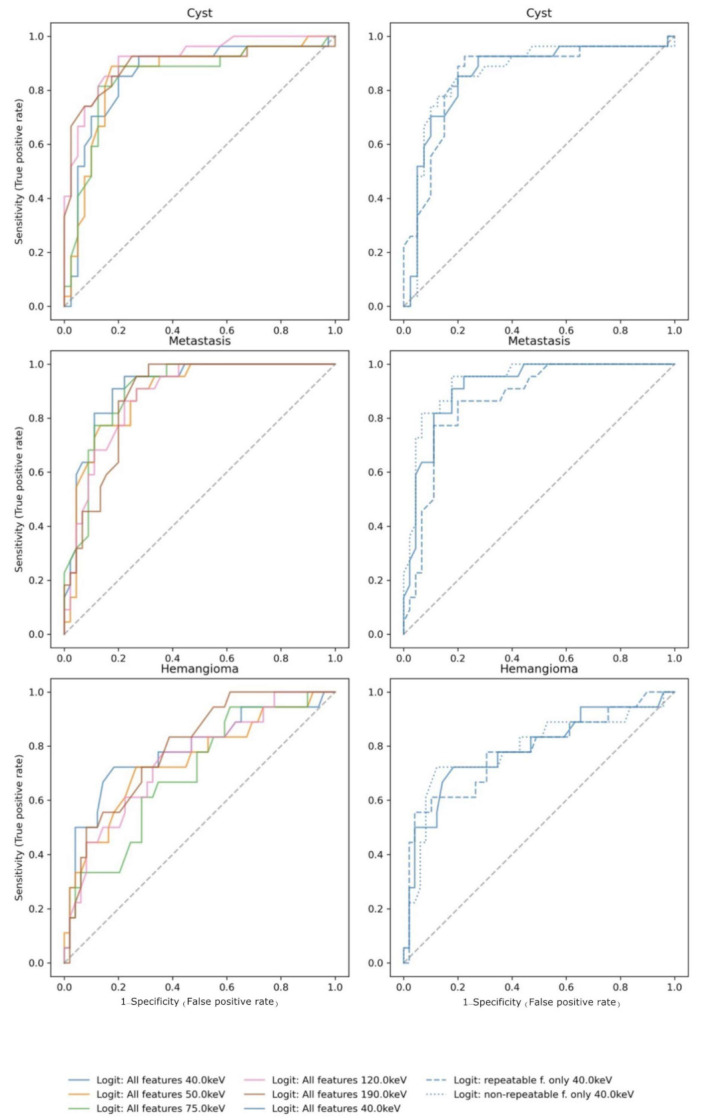
Receiver operating characteristics of the penalized logistic regression model for classification of cysts, metastases, and hemangioma by VMI energy (left column) and feature subset (right column), respectively. Color indicates VMI energy. Line style indicates the used feature subset (‘all features’, ‘repeatable only’, ‘nonrepeatable only’) at 40 keV.

The ROC curves for the different lesion entities (cysts, metastases, and hemangioma) are given for the penalized logistic regression model in Figure 5 by VMI energy (left column) and by feature subset for 40 keV VMI (right column). For classification of hemangioma and metastases, respectively, the best discrimination was achieved at lower VMI energies (40 keV), while discrimination of cysts was best at higher VMI energies (120 keV). Reducing the features to repeatable features only (CCC ≥ 0.9) did not improve classification performance. Interestingly, for metastases, the model containing only non-repeatable features showed the best discrimination at 40 keV. The corresponding calibration plots of the models are given in Supplementary Figures S3 and S4. For details on interpretation please refer to the figure legends.

Classification performance (AUC averaged over all classes) and prediction ((scaled) BS) for different VMI energies, feature subsets, and classification models are given in Table 2. AUC ranged between 0.77 and 0.86. Both the lowest prediction error and the best discrimination were achieved using the penalized logistic regression model with all radiomic features of the 120 keV images. Compared to a non-informative model (i.e., one that always predicts the training sample mean frequency) this model reduced the prediction error by 32% (R^2^).

Results from the sensitivity analysis (random forest models), also taking into account nonlinear effects and interactions, are documented in Table 2 and Supplementary Figures S4 and S5. Overall model performance and VMI energy dependence was similar to the penalized logistic regression model.

## 4. Discussion

In this study, we systematically assessed the test–retest repeatability and reproducibility of radiomic features derived from virtual monoenergetic images (VMI) of dual-energy CT (DECT) for different VMI energies, radiation doses, and DECT approaches ex vivo, and estimated their impact on machine-learning-based classification in a combined phantom and in vivo patient study.

The main results of our study can be summarized as follows: Overall, high feature repeatability was found, while reproducibility differed substantially among VMI energies within and between different DECT approaches. Despite these differences, classification performance was mainly unaffected by selecting only repeatable features. For the simple classification task in the phantom study, classification was nearly perfect when repeatable features were used and no differences by VMI energy, DECT, or radiation dose were observed. Here, classification performance suffered when relying solely on non-repeatable features. In vivo, optimal VMI energy differed among lesion types but overall classification performance was not improved by selecting repeatable features only.

### 4.1. Radiomic Feature Repeatability and Reproducibility

In the phantom part of our study, we demonstrated high test–retest repeatability within the same VMI energy for both DECT approaches with an average of 80% repeatable features across all test–retest experiments. Features from dual-source dual-energy (DSDE) VMI exhibited overall slightly higher stability and reproducibility compared to split-filter dual-energy (SFDE) VMI. Thus, the repeatability of most radiomic features appears to be independent of the individual VMI energy, while examinations performed on DSDE might be advantageous over SFDE for improving feature repeatability.

Despite these minor differences in repeatability, reproducibility dropped substantially if features derived from different VMIs were compared within the same DECT approach (for example, only 33% reproducible features comparing 40 and 190 keV in SFDS at 5 mGy). Interestingly, for both DECT approaches, the percentage of reproducible features decreased with increasing difference in VMI energy when comparing two VMIs (e.g., 32.8% for 40 keV vs. 190 keV and 98.4% for 120 keV vs. 190 keV for split-filter DECT at 5 mGy). Thus, in terms of reproducibility, different VMI energies should not be used interchangeably in radiomic studies, even if reconstructed from the same CT dataset. The potential additive value of different VMI energies for a clinical classification task has shown promising results in prior head and neck studies [15,16,17] and should thus be investigated further in other organs.

Similarly, our results regarding reproducibility between different DECT approaches show that results from different approaches within a (multicentric) study should be interpreted with caution, since reproducibility across DSDE and SFDE differed by VMI energy. Since features extracted from 75 keV VMIs showed the highest reproducibility across scanners, this VMI energy might be considered the most suitable for multi-scanner studies. 

Radiation dose did not show a relevant impact on reproducibility. Very high reproducibility was found for the two radiation doses within each DECT approach (>90%) except for VMI at 40 keV with a minimum of 81.9% for SFDE. Thus, radiomic analyses can even be performed in dose-optimized CT scans. This is of major clinical and scientific relevance, especially in the context of oncologic imaging, where patients receive multiple follow-up studies.

### 4.2. Impact on Machine Learning Classification

We also investigated the impact of different VMI energies and feature repeatability on machine-learning-based image classification for a simple classification task in the ex vivo study, and for a more sophisticated classification task in the in vivo study. 

In the phantom study, classification performance was nearly perfect when (at least) the repeatable features were presented to the learning algorithm and no differences by VMI energy, DECT, or radiation dose were observed. In absence of repeatable features (i.e., non-repeatable features only), classification performance decreased. For non-repeatable features, discrimination was still excellent (AUC ≥ 0.96 over all energies, doses, and DECT approaches), while calibration, and hence overall prediction error, worsened considerably (R^2^ was only 50% in the worst case, instead of nearly 100% when repeatable features were presented).

In the patient study, optimal classification performance depended on the VMI energy (AUC 0.77–0.86, R^2^ 0.17–0.32) and differed among lesion types (see ROC curves in Figure 5). For hemangioma and metastases, the best performance was achieved at low VMI energies, while for cysts the best performance was found at higher energies. We hypothesize that this difference might be due to the fact that hemangiomas and metastases have more areas of enhancing tissue after administration of iodinated contrast medium compared to cysts. These enhancing areas show a stronger increase in CT attenuation at low VMI energies (i.e., with increasing proximity to iodine’s K-edge). Interestingly, the best discrimination between metastasis and benign lesions was achieved at VMI energies which were different from the typical mean energy of a conventional single-energy CT at 120 kV (57 keV), thus underlining the potential information gain associated with using VMI of DECT.

In addition, the choice of feature subset (all features, repeatable features, or non-repeatable features) did not improve classification performance. There was a certain susceptibility to non-repeatable features mainly resulting in suboptimal calibration (i.e., the observed frequency did not match the predicted probability) and, hence, increased prediction error (i.e., the absolute error), while discrimination (i.e., whether the order of the classes is correct; that is, how often an object from a certain class has also a higher predicted probability for that class than a subject from other classes) mainly remained unaffected. For example, the classification of highest clinical relevance (metastasis vs. benign lesion) worked best with the subset of only non-repeatable features at 40 keV. This finding is in line with a study by Lv et al., in which poor repeatability of radiomic features did not necessarily translate into poor discrimination performance in PET-CT imaging [42]. Hence, discrepancies among the repeatability of radiomic features might be neglectable if an appropriate classification model is applied. Therefore, excluding features based on test–retest CCCs does not seem to result in optimal classification results. The present work indicates that non-repeatable features can still hold valuable information for classification and should therefore not be excluded solely due to a lack of repeatability.

We would like to emphasize that the main aim of the patient study was to investigate the impact of VMI energies and feature selection on differences in performance within classification models. Importantly, we did not aim to develop an accurate model for lesion classification. Nevertheless, for our sample, we found no evidence that classification could be improved by applying a learning algorithm that accounts for non-linear effects and interactions (random forest). When considering that only two classification algorithms were tested, and that the classification task was of low to intermediate difficulty in the patient study, future studies should further investigate this finding for different pathologies and machine learning classifiers to strengthen the use of VMI reconstructions in radiomics.

### 4.3. Study Limitations

First, we used a simplified texture model in our phantom study with a limited number of CT attenuation and texture differences, which do not reflect textures found in specific disease processes. Due to this simplified design, the classification task resulted in very good discrimination capabilities of all investigated reconstruction and scan parameters. Second, the limited sample size resulted in the choice of rather simple machine learning algorithms. The random forest especially, that also accounts for non-linear relations and interactions, would likely have profited from larger training samples. For a potential clinical application, the results of the machine-learning-based classification would need to be set in the context of a human reader. Here, the human reader provided the ground truth based on follow-up investigations and/or biopsies only, but was not used as a benchmark for classification. Third, the choice of CCC ≥ 0.9 as a cutoff for feature repeatability is arbitrary, although generally accepted. Fourth, even though we strictly separated training and test sets, while minimizing the pessimistic bias induced by the split procedure (by tournament-leave-pair-out cross-validation) and the optimism (by bootstrap-bias-correction) due to testing of multiple hyper-parameter configurations [15,17], the classification models were not externally validated. However, we would like to emphasize that this study aimed to investigate the impact of different VMI energies on classification, rather than to develop the best classification model where the differences in features across different energies might provide complementary information.

## 5. Conclusions

In an ex vivo setting, we observed a high test–retest repeatability of radiomic features from virtual monoenergetic images from dual-energy CT when keeping scan and reconstruction conditions constant. However, based on the finding of the diminished reproducibility of radiomics features when using different reconstruction energies or dual-energy CT approaches, different VMI energies should not be used interchangeably in radiomic studies, even if reconstructed from the same CT dataset. Features extracted from 75 keV VMIs showed the highest reproducibility across scanners and thus might be considered most suitable for multi-scanner studies. Since radiation dose did not show a relevant impact on reproducibility, radiomic analyses might be feasible in dose-optimized CT scans for multiple follow-up studies.

Classification performance was unaffected by the exclusion of non-repeatable features in vivo and ex vivo. The present work indicates that non-repeatable features can still hold valuable information for classification and should therefore not be excluded solely due to a lack of repeatability. The choice of VMI energy has the potential to improve lesion classification in vivo and should hence be adapted to the specific task. Moreover, differences in features across different energies might provide complementary information, which remains to be explored in future studies.

## Figures and Tables

**Table 1:** Study subjects.

Study Subjects (*n* = 72)	
Sex	33 male, 39 female
Age	66 ± 12 years
Final diagnoses	Lung cancer (*n* = 12) Colon cancer (*n* = 11) Kidney cancer (*n* = 9) Rectal cancer (*n* = 5) Sarcoma (*n* = 5) Leukemia (*n* = 4) Pancreatic cancer (*n* = 3) Breast cancer (*n* = 3) Peritoneal cancer (*n* = 3) Ovarian cancer (*n* = 3) Esophageal cancer (*n* = 2) Pleural mesothelioma (*n* = 2) Prostate cancer (*n* = 1) Gastrointestinal stromal tumor (*n* = 1) Stomach cancer (*n* = 1) Other (*n* = 7)

**Table 2:** In vivo classification performance for different machine learning models, feature selection strategies, and VMI energies.

Model	Features	Energy (keV)	AUC	Brier Score (BS)	R^2^ (Scaled BS)
Penalized Logistic Regression	all	40	0.84 (0.95; 0.72)	0.45 (0.29; 0.69)	0.29 (−0.11; 0.54)
		50	0.82 (0.93; 0.69)	0.48 (0.32; 0.73)	0.25 (−0.15; 0.49)
		75	0.80 (0.92; 0.70)	0.50 (0.33; 0.76)	0.22 (−0.18; 0.48)
		120	0.85 (0.94; 0.75)	0.43 (0.31; 0.57)	0.32 (0.11; 0.50)
		190	0.84 (0.94; 0.71)	0.45 (0.32; 0.63)	0.29 (0.026; 0.49)
	repeatable	40	0.81 (0.93; 0.68)	0.50 (0.34; 0.72)	0.22 (−0.13; 0.46)
		50	0.84 (0.94; 0.72)	0.45 (0.27; 0.65)	0.29 (−0.0076; 0.57)
		75	0.81 (0.92; 0.69)	0.50 (0.33; 0.69)	0.22 (−0.091; 0.48)
		120	0.79 (0.91; 0.67)	0.51 (0.36; 0.70)	0.21 (−0.095; 0.43)
		190	0.80 (0.91; 0.66)	0.51 (0.36; 0.70)	0.20 (−0.079; 0.42)
	non-repeatable	40	0.84 (0.96; 0.72)	0.45 (0.28; 0.71)	0.29 (−0.13; 0.55)
		50	0.81 (0.93; 0.68)	0.48 (0.33; 0.68)	0.25 (−0.063; 0.48)
		75	0.80 (0.92; 0.69)	0.50 (0.34; 0.74)	0.22 (−0.15; 0.46)
		120	0.84 (0.94; 0.73)	0.45 (0.33; 0.57)	0.30 (0.11; 0.48)
		190	0.83 (0.93; 0.70)	0.47 (0.34; 0.65)	0.27 (−0.0039; 0.46)
Random Forest	all	40	0.84 (0.94; 0.72)	0.45 (0.33; 0.58)	0.30 (0.086; 0.47)
		50	0.85 (0.94; 0.74)	0.45 (0.34; 0.59)	0.30 (0.089; 0.47)
		75	0.81 (0.92; 0.69)	0.48 (0.36; 0.62)	0.25 (0.04; 0.43)
		120	0.80 (0.92; 0.67)	0.49 (0.37; 0.63)	0.23 (0.026; 0.41)
		190	0.79 (0.91; 0.66)	0.50 (0.38; 0.64)	0.21 (0.016; 0.38)
	repeatable	40	0.83 (0.93; 0.72)	0.47 (0.35; 0.61)	0.27 (0.038; 0.46)
		50	0.82 (0.93; 0.71)	0.48 (0.36; 0.62)	0.25 (0.016; 0.43)
		75	0.80 (0.91; 0.68)	0.50 (0.37; 0.64)	0.22 (−0.0043; 0.41)
		120	0.79 (0.90; 0.66)	0.51 (0.37; 0.65)	0.20 (−0.0044; 0.39)
		190	0.77 (0.88; 0.64)	0.53 (0.41; 0.67)	0.17 (−0.037; 0.36)
	non-repeatable	40	0.85 (0.94; 0.74)	0.46 (0.35; 0.57)	0.29 (0.092; 0.44)
		50	0.86 (0.94; 0.75)	0.44 (0.34; 0.56)	0.31 (0.11; 0.46)
		75	0.82 (0.92; 0.72)	0.47 (0.36; 0.59)	0.26 (0.08; 0.42)
		120	0.81 (0.92; 0.68)	0.49 (0.38; 0.62)	0.24 (0.051; 0.4)
		190	0.81 (0.92; 0.68)	0.49 (0.38; 0.61)	0.23 (0.05; 0.39)

AUC = area under the receiver operating curve.

## Data Availability

The data presented in this study are available on request from the corresponding author.

## Supplementary Materials

The following are available online at https://www.mdpi.com/article/10.3390/cancers13184710/s1, Figure S1: Reproducibility of radiomic features between different dual-energy CT scanners, Figure S2: Reproducibility of radiomic features between different radiation doses, Figure S3: Calibration plot of the penalized logistic regression model for classification of cysts, metastases, and hemangioma, Figure S4: Calibration plot of the random forest model for classification of cysts, metastases, and hemangioma by VMI energy, Figure S5: Receiver operating characteristics of the random forest model for classification of cysts, metastases, and hemangioma, Table S1: In vitro classification performance metrics for penalized logistic regression by feature subset, DECT scanner, radiation dose, and VMI energy; Table S2: In-vitro classification performance metrics for penalized logistic regression by feature subset, DECT scanner, radiation dose, and VMI energy.
