# Supplementary material for: Virtual Monoenergetic Images of Dual-Energy CT—Impact on Repeatability, Reproducibility, and Classification in Radiomics
Source: Cancers (Basel). 2021 Sep 20;13(18):4710. doi: 10.3390/cancers13184710 (PMC8467875; doi:10.3390/cancers13184710)
Supplement: Supplementary file 1 [file cancers-13-04710-s001.zip › cancers-1289651-supplementary.pdf]

Supplemental Material for the Article:

Virtual Monoenergetic Images of Dual-Energy CT – Impact on Repeatability, Reproducibility, and Classification in Radiomics

André Euler, MD [1*], Fabian Christopher Laqua [2*], Davide Cester, PhD [3], Niklas Lohaus, MD [4], Thomas Sartoretti, BSc [5], Daniel Pinto dos Santos, MD [6], Hatem Alkadhi, MD, MPH, EBCR, FESER [7], and Bettina Baessler, MD [8]

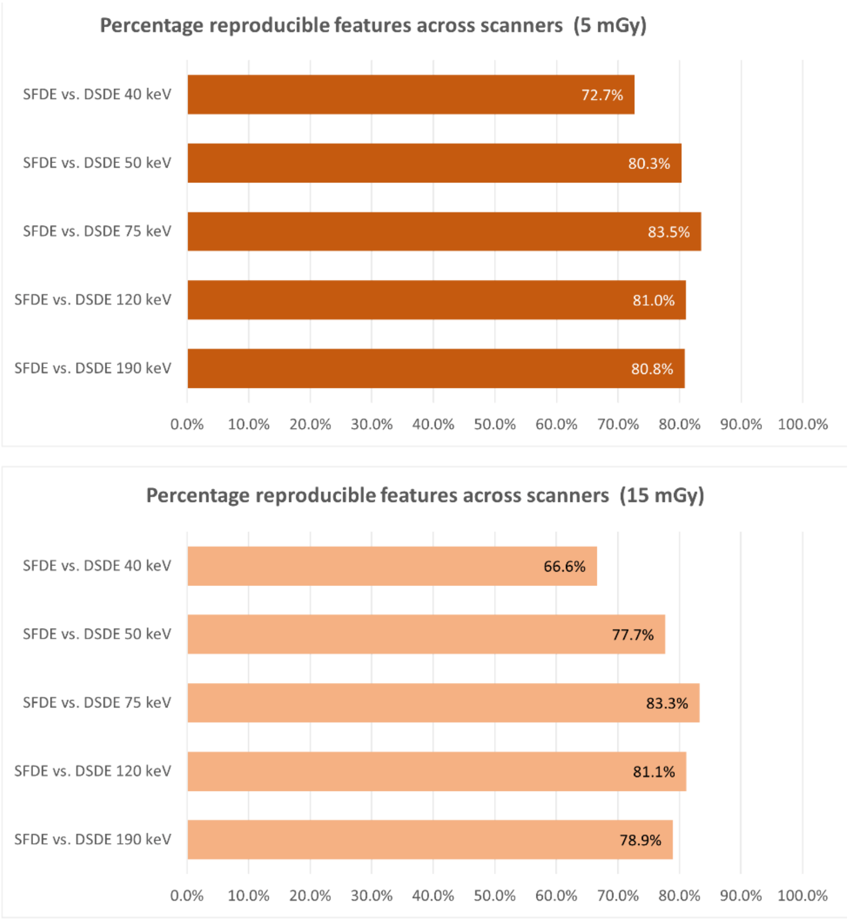

Supplementary Fig. S1: Reproducibility of radiomic features between different dual-energy CT scanners. Bar plots showing the percentage of reproducible features at equal VMI energies between SFDE and DSDE as a function of radiation dose. The highest percentage of reproducible features was achieved at VMI energies of 75 keV and the lowest at 40 keV.

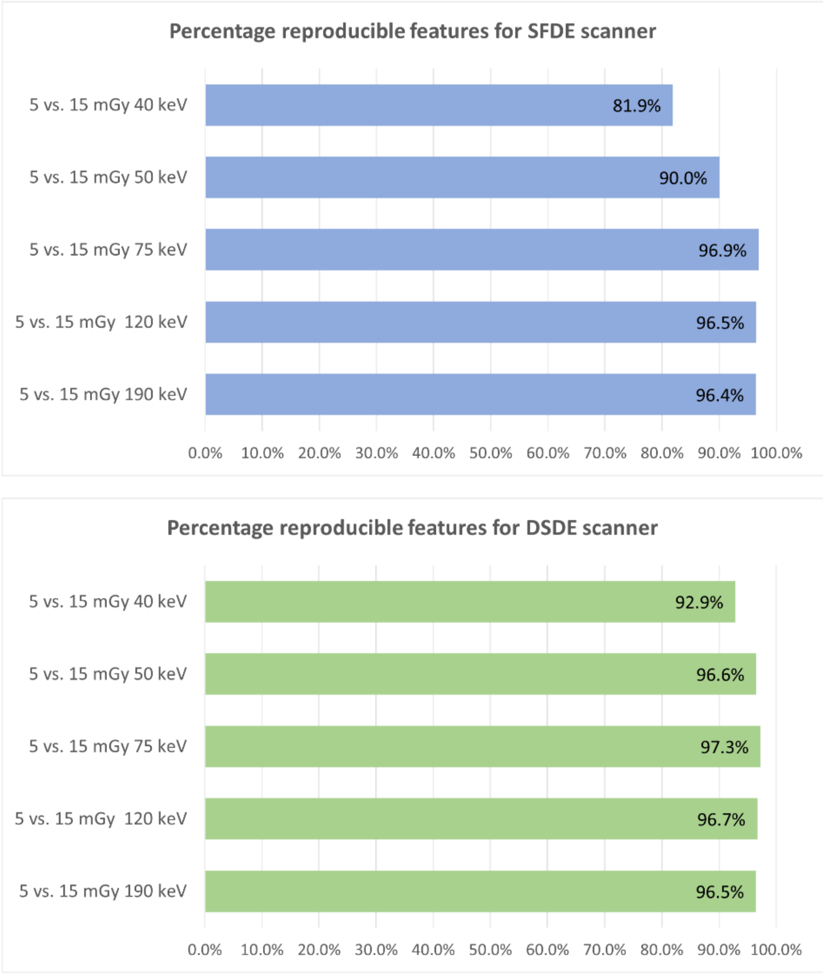

Supplementary Fig. S2: Reproducibility of radiomic features between different radiation doses
Bar plots showing the percentage of reproducible features at equal VMI energies between 5 and 15mGy for both DECT approaches. Overall, reproducibility was high, except for 40keV of SFDE. Highest reproducibility was achieved at 75 keV.

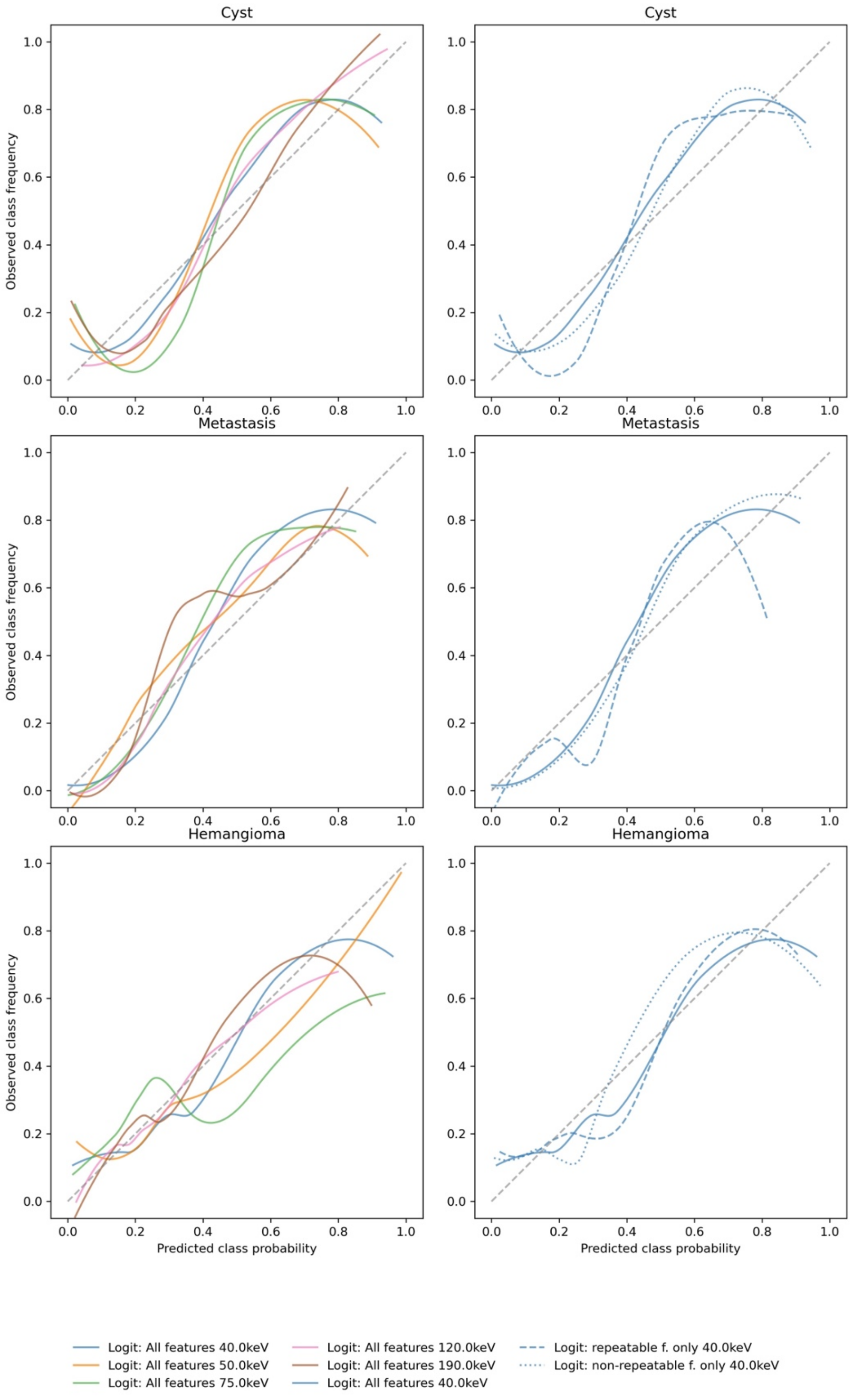

Supplementary Fig. S3: Calibration plot of the penalized logistic regression model for classification of cysts, metastases, and hemangioma by VMI energy (left column) and feature subset (right column), respectively. Color indicates VMI energy. Line style indicates used feature subset ('all features', 'repeatable only' 'non-repeatable only') at 40keV. For every class (hemangioma, cysts, and metastasis) the actual frequency of the occurrence (y-axis) is plotted over the predicted probability. Calibration is considered good when the curve is close to the diagonal. Departure to the top means that for predictions with this value the model underestimates the probability of an occurrence. Analog for departure to the bottom.

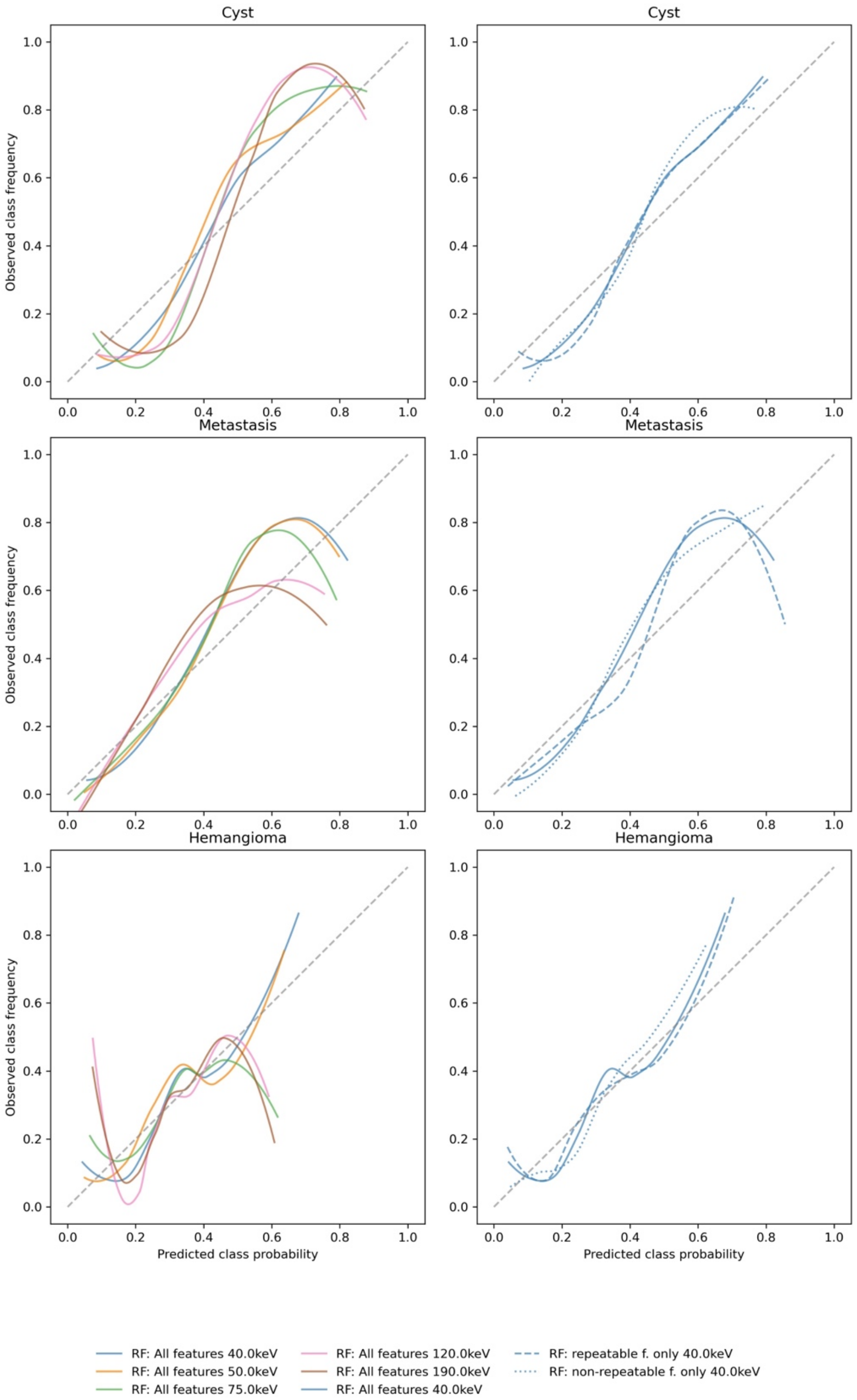

Supplementary Fig. S4: Calibration plot of the random forest model for classification of cysts, metastases, and hemangioma by VMI energy (left column) and feature subset (right column), respectively. Color indicates VMI energy. Line style indicates used feature subset ('all features', 'repeatable only' 'non-repeatable only') at 40keV. For every class (hemangioma, cysts, and metastasis) the actual frequency of the occurrence (y-axis) is plotted over the predicted probability. Calibration is considered good when the curve is close to the diagonal. Departure to the top means that for predictions with this value the model underestimates the probability of an occurrence. Analog for departure to the bottom.

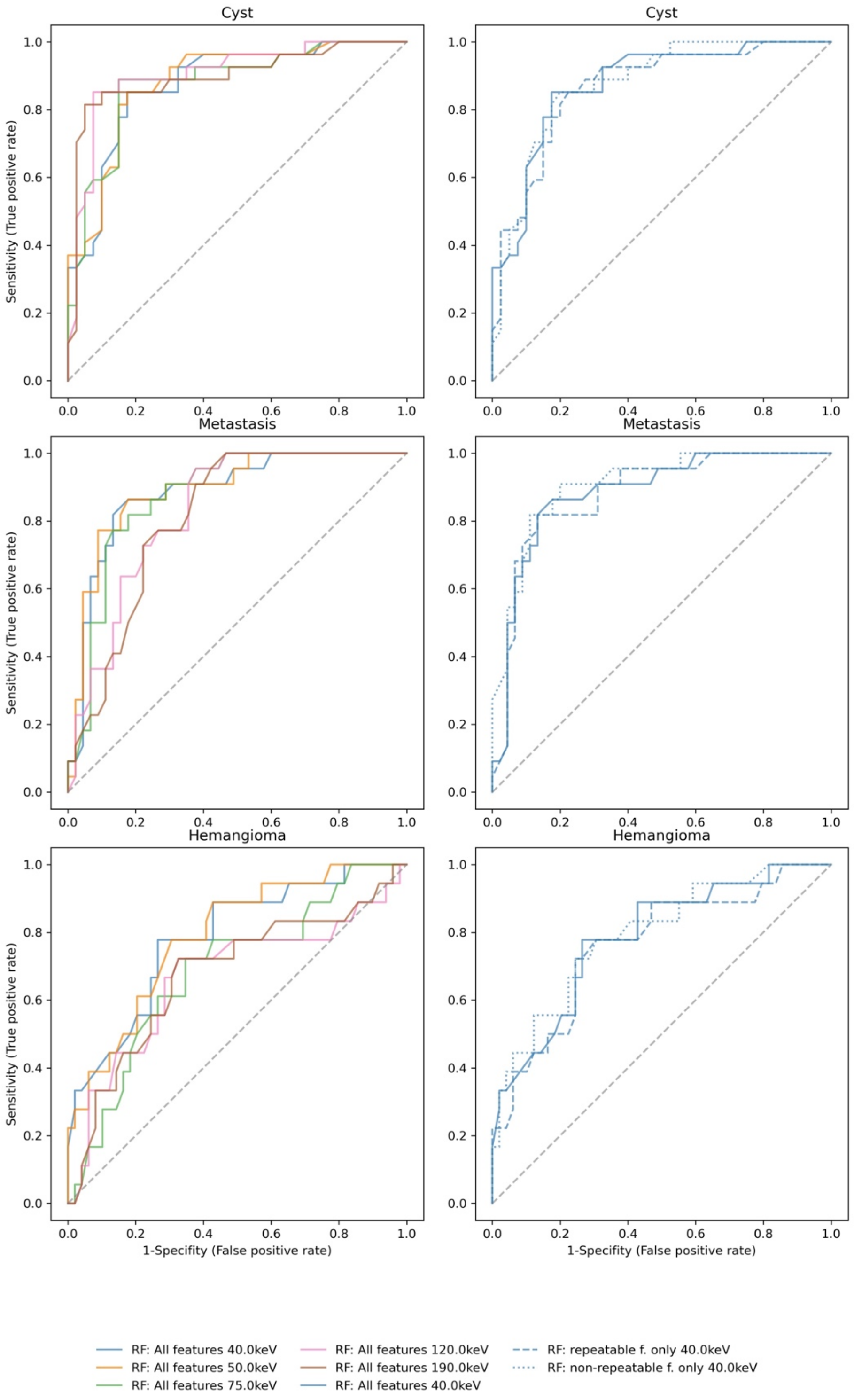

Supplementary Fig. S5: Receiver-operating-characteristics of the random forest model for classification of cysts, metastases, and hemangioma by VMI energy (left column) and feature subset (right column), respectively. Color indicates VMI energy. Line style indicates used feature subset ('all features', 'repeatable only' 'non-repeatable only') at 40keV.

Table S1: Extracted radiomic feature classes with corresponding feature names.

Feature family	Feature name
Intensity-based statistical (histogram) features	Mean (discretized) intensity<br>(Discretized) intensity variance<br>(Discretized) intensity skewness<br>(Excess) (discretized) intensity kurtosis<br>Median (discretized) intensity<br>Minimum (discretized) intensity<br>10th (discretized) intensity percentile<br>90th (discretized) intensity percentile<br>Maximum (discretized) intensity<br>(Discretized) intensity interquartile range<br>(Discretized) intensity range<br>(Discretized) intensity-based mean absolute deviation<br>(Discretized) intensity-based robust mean absolute deviation<br>(Discretized) intensity-based median absolute deviation<br>(Discretized) intensity-based coefficient of variation<br>(Discretized) intensity-based quartile coefficient of dispersion<br>(Discretized) intensity-based energy<br>Root mean square (discretized) intensity
Intensity-volume histogram features	Volume at intensity fraction<br>Intensity at volume fraction<br>Volume fraction difference between intensity fractions<br>Intensity fraction difference between volume fractions<br>Area under the IVH curve
Shape features	VoxelVolume<br>Maximum3DDiameter<br>MeshVolume<br>MajorAxisLength<br>Sphericity<br>LeastAxisLength<br>Elongation<br>SurfaceVolumeRatio<br>Maximum2DDiameterSlice<br>Flatness<br>SurfaceArea<br>MinorAxisLength<br>Maximum2DDiameterColumn<br>Maximum2DDiameterRow
Grey level co-occurrence matrix (GLCM)	Joint maximum<br>Joint average<br>Joint variance<br>Joint entropy<br>Difference average<br>Difference variance<br>Difference entropy<br>Sum average<br>Sum variance<br>Sum entropy<br>Angular second moment

	Contrast<br>Dissimilarity<br>Inverse difference<br>Normalized inverse difference<br>Inverse difference moment<br>Normalized inverse difference moment<br>Inverse variance<br>Correlation<br>Autocorrelation<br>Cluster tendency<br>Cluster shade<br>Cluster prominence<br>Information correlation 1<br>Information correlation 2
Grey level run length matrix (GLRLM)	Short runs emphasis<br>Long runs emphasis<br>Low grey level run emphasis<br>High grey level run emphasis<br>Short run low grey level emphasis<br>Short run high grey level emphasis<br>Long run low grey level emphasis<br>Long run high grey level emphasis<br>Grey level non-uniformity<br>Normalized grey level non-uniformity<br>Run length non-uniformity<br>Normalized run length non-uniformity<br>Run percentage<br>Grey level variance<br>Run length variance<br>Run entropy
Grey level size zone matrix (GLSZM)	Grey level size zone<br>Large zone emphasis<br>Low grey level zone emphasis<br>High grey level zone emphasis<br>Small zone low grey level emphasis<br>Small zone high grey level emphasis<br>Large zone low grey level emphasis<br>Large zone high grey level emphasis<br>Grey level non-uniformity<br>Normalized grey level non-uniformity<br>Zone size non-uniformity<br>Normalized zone size non-uniformity<br>Zone percentage<br>Grey level variance<br>Zone size variance<br>Zone size entropy
Grey level distance zone matrix (GLDZM)	Small distance emphasis<br>Large distance emphasis<br>Low grey level zone emphasis<br>High grey level zone emphasis<br>Small distance low grey level emphasis<br>Small distance high grey level emphasis<br>Large distance low grey level emphasis

	Large distance high grey level emphasis<br>Grey level non-uniformity<br>Normalized grey level non-uniformity<br>Zone distance non-uniformity<br>Normalized zone distance non-uniformity<br>Zone percentage<br>Grey level variance<br>Zone distance variance<br>Zone distance entropy
Neighborhood grey tone difference matrix (NGTDM)	Coarseness<br><br>Contrast<br>Busyness<br>Complexity<br>Strength
Neighboring grey level dependence matrix (NGLDM)	Low dependence emphasis<br><br>High dependence emphasis<br>Low grey level count emphasis<br>High grey level count emphasis<br>Low dependence low grey level emphasis<br>Low dependence high grey level emphasis<br>High dependence low grey level emphasis<br>High dependence high grey level emphasis<br>Grey level non-uniformity<br>Normalized grey level non-uniformity<br>Dependence count non-uniformity<br>Normalized dependence count non-uniformity<br>Dependence count percentage<br>Grey level variance<br>Dependence count variance<br>Dependence count entropy<br>Dependence count energy

Each feature was extracted as an original feature as well as using wavelet transform in 8 decompositions and Laplacian of Gaussian filters with sigma values from 1 to 5. For mathematical definitions of all features, please refer to the recommendations of the Image Biomarker Standardization Initiative (IBSI) (Zwanenburg et al., 2020, 2016)

Table S2: In-vitro classification performance metrics for penalized logistic regression by feature subset, DECT scanner, radiation dose, and VMI energy.

Features	Scanner	Dose [mGy]	Energy [keV]	AUC	Brier score (BS)	R^2 (scaled BS)
all	DSDE	5	40	1.0 (1.0;1.0)	0.00024 (6e-07;0.00092)	1.0 (1.0;1.0)
			50	1.0 (1.0;1.0)	0.0002 (5.3e-07;0.00073)	1.0 (1.0;1.0)
			75	1.0 (1.0;1.0)	0.00019 (5.4e-07;0.00068)	1.0 (1.0;1.0)
			120	1.0 (1.0;1.0)	0.00013 (5.8e-07;0.00045)	1.0 (1.0;1.0)
			190	1.0 (1.0;1.0)	0.00013 (4.6e-07;0.00045)	1.0 (1.0;1.0)
		15	40	1.0 (1.0;1.0)	0.0001 (3.6e-07;0.00034)	1.0 (1.0;1.0)
			50	1.0 (1.0;1.0)	0.00011 (3.4e-07;0.00036)	1.0 (1.0;1.0)
			75	1.0 (1.0;1.0)	0.00012 (5.8e-07;0.00042)	1.0 (1.0;1.0)
			120	1.0 (1.0;1.0)	0.00013 (5.2e-07;0.00044)	1.0 (1.0;1.0)
			190	1.0 (1.0;1.0)	0.00012 (8.5e-07;0.0004)	1.0 (1.0;1.0)
	SFDE	5	40	1.0 (1.0;1.0)	0.00024 (6.3e-07;0.00087)	1.0 (1.0;1.0)
			50	1.0 (1.0;1.0)	0.00022 (5.1e-07;0.00084)	1.0 (1.0;1.0)
			75	1.0 (1.0;1.0)	0.00019 (4.6e-07;0.00071)	1.0 (1.0;1.0)
			120	1.0 (1.0;1.0)	0.00021 (3.9e-07;0.00077)	1.0 (1.0;1.0)
			190	1.0 (1.0;1.0)	0.00019 (3.7e-07;0.00066)	1.0 (1.0;1.0)
		15	40	1.0 (1.0;1.0)	0.00014 (6.9e-07;0.0005)	1.0 (1.0;1.0)
			50	1.0 (1.0;1.0)	0.00012 (7.1e-07;0.00043)	1.0 (1.0;1.0)
			75	1.0 (1.0;1.0)	0.00016 (5.1e-07;0.00055)	1.0 (1.0;1.0)
			120	1.0 (1.0;1.0)	0.00015 (3.4e-07;0.00054)	1.0 (1.0;1.0)
			190	1.0 (1.0;1.0)	0.00017 (4.9e-07;0.0006)	1.0 (1.0;1.0)
repeatable	DSDE	5	40	1.0 (1.0;1.0)	0.00012 (1.4e-07;0.00045)	1.0 (1.0;1.0)
			50	1.0 (1.0;1.0)	0.00014 (1.6e-07;0.00054)	1.0 (1.0;1.0)
			75	1.0 (1.0;1.0)	9.7e-05 (1.4e-07;0.00036)	1.0 (1.0;1.0)
			120	1.0 (1.0;1.0)	8.1e-05 (1.7e-07;0.0003)	1.0 (1.0;1.0)
			190	1.0 (1.0;1.0)	8.5e-05 (1.7e-07;0.00032)	1.0 (1.0;1.0)
		15	40	1.0 (1.0;1.0)	0.00015 (3.2e-07;0.00055)	1.0 (1.0;1.0)
			50	1.0 (1.0;1.0)	0.00014 (2.7e-07;0.00051)	1.0 (1.0;1.0)
			75	1.0 (1.0;1.0)	0.00013 (3.4e-07;0.00048)	1.0 (1.0;1.0)

			120	1.0 (1.0;1.0)	0.00015 (4e-07;0.00054)	1.0 (1.0;1.0)
			190	1.0 (1.0;1.0)	0.00012 (4.3e-07;0.00046)	1.0 (1.0;1.0)
	SFDE	5	40	1.0 (1.0;1.0)	0.00022 (1.3e-07;0.00082)	1.0 (1.0;1.0)
			50	1.0 (1.0;1.0)	0.00019 (1.5e-07;0.00075)	1.0 (1.0;1.0)
			75	1.0 (1.0;1.0)	0.00012 (1.5e-07;0.00046)	1.0 (1.0;1.0)
			120	1.0 (1.0;1.0)	0.00011 (1.7e-07;0.00043)	1.0 (1.0;1.0)
			190	1.0 (1.0;1.0)	0.00011 (1.4e-07;0.00042)	1.0 (1.0;1.0)
		15	40	1.0 (1.0;1.0)	0.00018 (1.5e-07;0.00061)	1.0 (1.0;1.0)
			50	1.0 (1.0;1.0)	0.00011 (1.2e-07;0.0004)	1.0 (1.0;1.0)
			75	1.0 (1.0;1.0)	0.00012 (1.3e-07;0.00045)	1.0 (1.0;1.0)
			120	1.0 (1.0;1.0)	0.00013 (1.5e-07;0.00048)	1.0 (1.0;1.0)
			190	1.0 (1.0;1.0)	0.00014 (1.5e-07;0.00052)	1.0 (1.0;1.0)
non-repeatable	DSDE	5	40	0.99 (1.0;0.9)	0.11 (0.011;0.31)	0.83 (0.5;0.98)
			50	1.0 (1.0;0.97)	0.066 (0.004;0.17)	0.89 (0.71;0.99)
			75	1.0 (1.0;1.0)	0.04 (0.0025;0.11)	0.93 (0.81;1.0)
			120	1.0 (1.0;1.0)	0.022 (0.0013;0.051)	0.96 (0.91;1.0)
			190	1.0 (1.0;1.0)	0.034 (0.0015;0.11)	0.94 (0.81;1.0)
		15	40	1.0 (1.0;0.96)	0.092 (0.00069;0.28)	0.85 (0.48;1.0)
			50	1.0 (1.0;0.99)	0.054 (0.0015;0.12)	0.91 (0.79;1.0)
			75	1.0 (1.0;0.96)	0.15 (0.00085;0.4)	0.76 (0.31;1.0)
			120	0.99 (1.0;0.94)	0.14 (0.003;0.42)	0.78 (0.33;0.99)
			190	0.98 (1.0;0.88)	0.25 (0.0051;0.64)	0.61 (-0.0022;0.99)
	SFDE	5	40	1.0 (1.0;1.0)	0.011 (0.00022;0.028)	0.98 (0.91;1.0)
			50	0.99 (1.0;0.9)	0.15 (0.015;0.44)	0.77 (0.25;0.98)
			75	0.98 (1.0;0.86)	0.19 (0.0043;0.56)	0.7 (0.0;0.99)
			120	0.99 (1.0;0.89)	0.17 (0.013;0.59)	0.73 (0.092;0.98)
			190	1.0 (1.0;0.96)	0.11 (0.0021;0.48)	0.82 (0.25;1.0)
		15	40	1.0 (1.0;1.0)	0.044 (0.00027;0.34)	0.93 (0.49;1.0)
			50	0.98 (1.0;0.8)	0.18 (0.011;0.69)	0.72 (0.01;0.98)
			75	0.96 (1.0;0.74)	0.31 (0.031;0.67)	0.52 (-0.11;0.94)

	120	0.98 (1.0;0.86)	0.2 (0.013;0.52)	0.69 (0.074;0.98)
	190	0.98 (1.0;0.86)	0.17 (0.02;0.49)	0.73 (0.13;0.97)
